# Association Between Religiosity and Spirituality and Cocaine Use: A Systematic Review

**DOI:** 10.1007/s10943-024-02069-6

**Published:** 2024-06-10

**Authors:** Alessandra Buja, Laura Montecchio, Francesca Dossi

**Affiliations:** Department of Cardiologic, Vascular and Thoracic Sciences, and Public Health, University of Padua, Padua, Italy

**Keywords:** Religiosity, Spirituality, Cocaine, Abuse

## Abstract

Previous literature has evidenced a possible impact on health, mental health, and health-related faith behaviors due to the effects of an individual’s spiritual dimension. The aim of this study is to collect and summarize all current data from observational studies regarding the association between religiosity or spirituality (R/S) and cocaine use (CU). A systematic literature search of analytical observational studies on the association between religiosity or spirituality and the use of cocaine was performed in PubMed and Scopus databases. Twenty observational studies were included in this review. Fifteen of the twenty observational studies found that a higher level of religiosity was associated with lower lifetime and actual cocaine use, both in adults and adolescents. However, one study conducted in a sexual minorities sample found that higher religiosity—measured as frequency of private religious activities such as prayer—was associated with a higher probability of cocaine use. Two studies found no evidence of any association between religiosity and cocaine use, and two found mixed results. This review found a possible protective role of religiosity on cocaine use, even if the cross-sectional nature of the greater part of the studies prevented drawing any casual relation. Future studies with a longitudinal approach are required. However, the support of activities aimed at broadening a religious attitude and beliefs could result in creating an environment protective for young people against cocaine use.

## Introduction

Religion typically refers to organized systems of beliefs, practices, rituals, and a community of followers. Spirituality, on the other hand, is a more personal and individualistic concept. It relates to the inner search for meaning, purpose, and connection to something greater than oneself, but it doesn’t necessarily adhere to any specific religious doctrine or institution. Religiosity is an aspect that has accompanied humanity since its inception, and in 2022 about 85% of the world’s people identify with a religion (Religion by Country, 2022). There is a lively medical–scientific interest in studying the possible impact on health, mental health, and on health-related behaviors attributable to the profession of a faith or having one’s spiritual dimension (Hodapp & Zwingmann, 2019; Koenig, 2009; Weber & Pargament, 2014). The mechanisms by which religion acts on health-related behavior have not yet been fully clarified; however, several studies have shown that the role of social support that religion provides to the faithful is central: participating in religious activities in the community creates important bonds between individuals who share values and interests, ensuring practical and emotional support (Kodzi et al., 2011; Koenig et al., 1997). Also, the salutogenic behavioral indications that many religions convey play an important role; acting as factors contributing to the promotion and maintenance of physical and mental well-being (Hill et al., 2007; Strawbridge et al., 2001). Finally, a series of psychosocial factors such as the provision of a meaningful sense of life, the reduction in stress, and the provision of coping mechanisms contribute to the influence that religiosity and spirituality exert on health behavior (Ahmadi et al., 2019; Krause, 2006; Pargament et al., 2000).

In particular, the impact of (religiosity and spirituality) R/S on substance use-related behaviors (Dodor et al., 2018; Kub & Solari-Twadell, 2013; Livne et al., 2021) has received attention in recent years; substance use is a growing health and social issue, affecting the whole world (Connery et al., 2020; Hall et al., 2016). In 2016, it was estimated that the drug-attributable disease burden accounted for approximately two percent of the global burden of disease overall (GBD, 2016 Alcohol & Drug Use Collaborators, 2018). In addition, substance use disorders contribute to additional morbidity and mortality because of the association with other medical conditions such as infectious diseases, including Hepatitis B, C, and HIV, and cardiovascular diseases (Degenhardt & Hall, 2012; Degenhardt et al., 2017). Specifically, the use of cocaine (and its derivatives, such as crack), a powerful central nervous system stimulant, generates important adverse health effects, including acute toxic effects, dependence, cardiovascular disease, and alterations in cognitive functioning (Butler et al., 2017; Frazer et al., 2018); cocaine use also has repercussions at the social level in terms of increased criminal behavior (Bennett et al., 2008). In 2021, in Europe, 4.8% of the population between 15 and 64 years reported lifetime use of cocaine, and 1.2% reported use in the last year (share of drug use in the European Union by type of drug, 2021). Nowadays, there are still no pharmacological treatments of safe and proven effectiveness for cocaine use disorder, and psychosocial treatments tend to have high relapse rates (Kampman, 2019). Consequently, identifying the protective and risk factors for the use of substances is of vital importance to address this phenomenon and stem it, possibly implementing programs and interventions aimed especially at the population groups exposed to risk factors or trying to increase exposure to protective factors.

We conducted a systematic review of observational studies evaluating the association between R/S and cocaine use to shed more light on this topic.

## Methods

### Search Strategy

For the present study, a comprehensive and systematic literature search was conducted in the PubMed and Scopus databases to identify observational studies (cross-sectional, cohort, and case–control studies) investigating the association between R/S and cocaine use.

The search process involved using a search string obtained by combining the terms “religiosity,” “spirituality,” “religion,” “faith,” or “religiousness” with the terms “problem*,” “addict*,” “compulsive,” “abuse*,” “dependen*,” “disorder*,” “use,” “substance*,” “drug*,” “patholog*,” and “cocaine” using Boolean operators. The search strings are reported in Table 1. The records retrieved from the databases were imported into EndNote, and duplicates were removed. The reviewer checked the search hits by reading the article titles and abstracts. If the results of a study were published more than once, only the most complete article was considered in the analysis. The authors also checked the reference lists of the papers included in the review for any articles not already considered.

**Table 1:** Boolean search strings applied in different databases

*Pubmed search string:*	(Religion[Title/Abstract] OR spirituality [Title/Abstract] OR religiosity[Title/Abstract] OR faith[Title/Abstract] OR religiousness[Title/Abstract]) AND (patholog*[Title/Abstract] OR problem*[Title/Abstract] OR addict*[Title/Abstract] OR compulsive[Title/Abstract] OR abuse*[Title/Abstract] OR dependen*[Title/Abstract] OR disorder*[Title/Abstract] OR use[Title/Abstract]) AND (substance*[Title/Abstract] OR drug*[Title/Abstract] OR cocaine[Title/Abstract]) AND English[lang] AND ((“1900/01/01”[PDAT]: “2021/11/24”[PDAT])
*Scopus search string:*	(TITLE-ABS (religion) OR TITLE-ABS (spirituality) OR TITLE-ABS (religiosity) OR TITLE-ABS (faith) OR TITLE-ABS (religiousness)) AND (TITLE-ABS (patholog*) OR TITLE-ABS (problem*) OR TITLE-ABS (addict*) OR TITLE-ABS (compulsive) OR TITLE-ABS (dependen*) OR TITLE-ABS (disorder*) OR TITLE-ABS (abuse*) OR TITLE-ABS (use*)) AND (TITLE-ABS (substance*) OR TITLE-ABS (drug*) OR TITLE-ABS (cocaine)) AND (LIMIT-TO (LANGUAGE, “English”)) AND PUBYEAR < 2022

### Data Extraction

The following data were extracted from each study: the first author’s name, year of publication, journal, study design, sampling method, characteristics of the study sample (e.g., age range), measures of outcome and exposure, results, confounding factors, interactions, and the author’s conclusions..

### Eligibility Criteria

The studies included in the review had to meet the following inclusion criteria:

- Reporting a declared measure used to evaluate religiosity or spirituality—considering spirituality as an aspect that can be experienced both outside and inside a religious context (Benson et al., 2003) and characterized by a desire for transcendence, a sense of interconnection, and a meaningful sense of life (King & Boyatzis, 2015).
- Reporting a measure of the association between religiosity/spirituality and cocaine use.
- Published up until December 2021 (for Scopus), or November 2021 (for Pubmed),
- Written in English.

The studies excluded in the review were those:

- Involving only selected samples of people using substances,
- Involving only sample with a declared belonging to a faith.
- Experimental studies regarding intervention aimed to quitting cocaine use.

### Methodological Assessment

An author judged the methodological appropriateness of the studies using the Strengthening the Reporting of Observational Studies in Epidemiology (STROBE) approach (von Elm et al., 2008). For observational studies, the total STROBE score was calculated for each study. A larger percentage of items conforming to the guidelines indicated higher methodological completeness: ≥ 80% excellent completeness; 60–79% good completeness; 50–59% sufficient completeness; < 50% poor completeness. The majority of the studies included in this systematic review had a score above good completeness. Table 2 outlines the methodological appropriateness of the studies.

## Results

The reference lists of the 18 selected articles yielded 2 additional papers meeting our inclusion and exclusion criteria. The review was thus conducted on 20 papers, and all were observational studies; eighteen of them were cross-sectional (Allen & Lo, 2010; Brown et al., 2001; Degenhardt et al., 2007; Dunn, 2005; Engs & Mullen, 1999; Gmel et al., 2013; Grunbaum et al., 2000; Lamb et al., 2019; Miller et al., 2000; Nicholson & Ford, 2019; Palamar & Ompad, 2014; Palamar et al., 2013; Salas-Wright et al., 2012, 2015; Wallace & Bachman, 1991; Watkins et al., 2016; Wray-Lake et al., 2012; Zanetti et al., 2019) and two longitudinal studies (Fletcher et al., 2014; Fothergill et al., 2009).

**Fig. 1:**
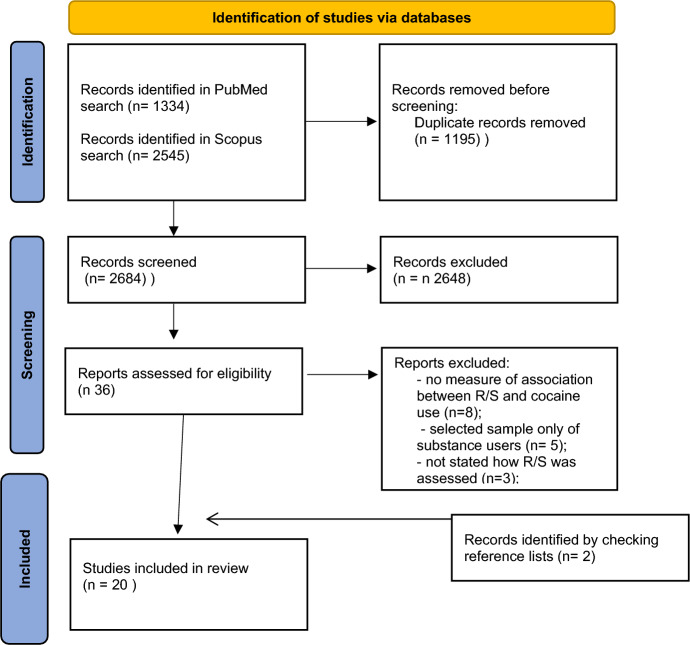
PRISMA flowchart: religion, spirituality (R/S) and cocaine use (CU)

Figure 1 shows the PRISMA flow diagram of the article selection process.

Considering these 20 articles, the number of participants enrolled in each observational study ranged from 151 to 188,682. Eleven studies (Allen & Lo, 2010; Degenhardt et al., 2007; Engs & Mullen, 1999; Fothergill et al., 2009; Gmel et al., 2013; Lamb et al., 2019; Nicholson & Ford, 2019; Palamar et al., 2013; Salas-Wright et al., 2015; Watkins et al., 2016; Zanetti et al., 2019) were conducted on adults, two studies on adolescents—in one study the sample was between 15 and 18 years old (Miller et al., 2000), while in the other it was between 12 and 17 years old (Salas-Wright et al., 2012)—and in seven studies on mixed samples (typically high school seniors who were both above and under 18 years of age (Brown et al., 2001; Dunn, 2005; Fletcher et al., 2014; Grunbaum et al., 2000; Palamar & Ompad, 2014; Wallace & Bachman, 1991; Wray-Lake et al., 2012).

The studies were conducted most in the USA (17 studies), but also in Brazil (Zanetti et al., 2019), Scotland (Engs & Mullen, 1999), and Switzerland (Gmel et al., 2013), and were published between 1991 and 2019.

Religiosity was often measured using single-item or two-item questionnaires, which included inquiries about the frequency of attendance at worship services and the importance or influence of religion in one’s life. Specifically, two studies utilized only a question about worship frequency to assess religiosity (Fothergill et al., 2009; Grunbaum et al., 2000), while four studies focused solely on the importance of religion (Degenhardt et al., 2007; Dunn, 2005; Engs & Mullen, 1999; Zanetti et al., 2019). One study assessed the influence of religious beliefs on decision-making in one’s life using a single question (Nicholson & Ford, 2019). Additionally, six studies employed questions regarding both the importance of religion in one’s life and worship attendance (Brown et al., 2001; Fletcher et al., 2014; Palamar & Ompad, 2014; Palamar et al., 2013; Wallace & Bachman, 1991; Wray-Lake et al., 2012). About the other seven studies, one used a subscale of the Duke University Religion Index (DUREL) (Lamb et al., 2019) to assess religiosity; one study used the first question of the Religious Background and Behavior Questionnaire (Gmel et al., 2013); one used an ad hoc four-items questionnaire (Salas-Wright et al., 2015); one used an ad hoc seven-items questionnaire by which a personal devotion score was assessed (Miller et al., 2000); one study used an ad hoc five-item questionnaire (Salas-Wright et al., 2012); one created a religiosity index with an ad hoc three-item questionnaire and a spirituality index with an ad hoc eleven-items questionnaire (Allen & Lo, 2010); and a last study created a religiosity index with an ad hoc four-item questionnaire and a spirituality index with a three-item questionnaire (Watkins et al., 2016)—and just the last two mentioned studies explored the sphere of spirituality.

Just two studies therefore used questions from validated questionnaires created to assess religiosity, and in general emerged some variabilities in how exposure was measured; only five studies asked about the type of religion of participants (if they have one); all the others did not investigate this aspect.

A total of twenty studies varied even in their approach to measuring the outcome—cocaine use—all based on self-reported information. In the majority of cases, participants answered questions about their lifetime use of cocaine (7 studies: Allen & Lo, 2010; Degenhardt et al., 2007; Dunn, 2005; Palamar & Ompad, 2014; Palamar et al., 2013; Watkins et al., 2016; Wray-Lake et al., 2012) or past-year use of cocaine (7 studies: Brown et al., 2001; Engs & Mullen, 1999; Gmel et al., 2013; Nicholson & Ford, 2019; Salas-Wright et al., 2012, 2015; Wallace & Bachman, 1991), with one of them using The Queensland Alcohol and Drug Study Questionnaire (Engs & Mullen, 1999). One study asked about both lifetime and past-year use of cocaine (Miller et al., 2000), while another asked about lifetime and last three months use of cocaine (Zanetti et al., 2019). Two studies asked about last month use of cocaine (Grunbaum et al., 2000; Lamb et al., 2019), with one of these studies using questions from the Youth Risk Behavior Survey (Grunbaum et al., 2000). One study asked about last 10 years use of cocaine (Fothergill et al., 2009), and one asked if participants have used cocaine since 1995, the year of the beginning of data collection in that longitudinal study (Fletcher et al., 2014).

Tables 3 and 4 provide details of the observational studies measuring only religiosity identified and included in the review, while Tables 3 and 6 provide details of the observational studies measuring both religiosity and spirituality.

Of the 20 included studies, fifteen found an association between higher religiosity and no cocaine use (Brown et al., 2001; Degenhardt et al., 2007; Dunn, 2005; Fothergill et al., 2009; Gmel et al., 2013; Grunbaum et al., 2000; Miller et al., 2000; Nicholson & Ford, 2019; Palamar & Ompad, 2014; Palamar et al., 2013; Salas-Wright et al., 2012, 2015; Wallace & Bachman, 1991; Wray-Lake et al., 2012; Zanetti et al., 2019), one found a connection between higher religiosity–in that study intended as frequency of private religious activities such as prayer—and cocaine use (Lamb et al., 2019), two found no evidence of any association between religiosity and cocaine use (Engs & Mullen, 1999; Fletcher et al., 2014), and two found mixed results: in Allen and Lo (2010) having higher spirituality was associated with cocaine use, while having higher religiosity was a protective factor for cocaine use; in Watkins et al. (2016) having higher spirituality was associated with no cocaine use, while having higher religiosity was a risk factor for cocaine use.

A total of fifteen studies found an association between higher religiosity and lower cocaine use, with seven conducted only on adults (Degenhardt et al., 2007; Fothergill et al., 2009; Gmel et al., 2013; Nicholson & Ford, 2019; Palamar et al., 2013; Salas-Wright et al., 2015; Zanetti et al., 2019), two conducted only on adolescents (Miller et al., 2000; Salas-Wright et al., 2012), and six conducted on both adults and adolescents (Brown et al., 2001; Dunn, 2005; Grunbaum et al., 2000; Palamar & Ompad, 2014; Wallace & Bachman, 1991; Wray-Lake et al., 2012). Thirteen of these studies were conducted in the USA (Brown et al., 2001; Degenhardt et al., 2007; Dunn, 2005; Fothergill et al., 2009; Grunbaum et al., 2000; Miller et al., 2000; Nicholson & Ford, 2019; Palamar & Ompad, 2014; Palamar et al., 2013; Salas-Wright et al., 2012, 2015; Wallace & Bachman, 1991; Wray-Lake et al., 2012) and two in other countries, Brazil and Switzerland (Gmel et al., 2013; Zanetti et al., 2019).

Five of these studies were conducted using US nationally representative data on samples of high school seniors (Brown et al., 2001; Dunn, 2005; Palamar & Ompad, 2014; Wallace & Bachman, 1991; Wray-Lake et al., 2012), technically mixed samples between adolescents and adults, but the latter were considered “emerging adults.” A specific group just over 18 years old was also investigated by Palamar et al. (2013), highlighting an association between never cocaine users and high religiosity. Both Miller et al. (2000) and Salas-Wright et al. (2012) conducted their studies using nationally representative data on adolescents, one with a sample between 15 and 18 years old and the other between 12 and 17 years old, showing an inverse association between religiosity and cocaine use. In particular, Salas-Wright et al. (2012) used an ad hoc five-item questionnaire to assess religiosity, identifying five different profiles of adolescent religiosity and showing a protective association of being part of a moderate or high religiosity class; Miller et al. (2000) used an ad hoc seven-item questionnaire to assess a personal devotion score, showing a protective role of a higher score.

Among the studies conducted on adults showing an inverse association between religiosity and cocaine use, Gmel et al. (2013) studied only men visiting recruitment centers in Switzerland, and Zanetti et al. (2019) selected samples of university students. Moreover, these two studies conducted outside the USA focused on particular population targets, namely young men about to begin military service and university students. All of the studies finding an association between higher Religiosity and no cocaine use did not select samples based on ethnicity, except for Nicholson and Ford (2019) and Fothergill et al. (2009), who selected only Black people in their samples. The study by Fletcher et al. (2014) was conducted on high school seniors in the USA, with a sample of around 15,000 students. The results showed that both the importance of religion and attendance at worship services reduced the propensity to use cocaine, but the estimates were not statistically significant. Similarly, the study by Engs and Mullen (1999), conducted on university students in Scotland, showed no significant difference between higher or lower religiosity and the use of cocaine. Allen and Lo (2010), in their study conducted on adults in the USA, showed mixed results about R/S and cocaine use. Here, having high religiosity was protective against cocaine use, consistent with the 15 studies mentioned earlier. However, they also found that high spirituality was associated with cocaine use.

Finally, two studies were conducted on samples with specific characteristics: one included only men of Latino/Hispanic ethnicity identifying as gay or bisexual (Lamb et al., 2019), while the other included only Black men sexually active with another man within the past year (Watkins et al., 2016). The first found higher cocaine use among participants with higher religiosity (here intended as the frequency of private religious activities such as prayer), while the second found that high spirituality was associated with no cocaine use, while high religiosity was a risk factor for cocaine use.

## Discussion

A total of fifteen out of the twenty observational studies found that a higher level of religiosity was associated with lower lifetime and past-year cocaine use (CU), indicating that religiosity is a determinant of the use of this specific substance, both in adults and adolescents.

Only two studies explored the connection between spirituality and cocaine use, yielding opposite results: one found an association between higher spirituality and lower lifetime cocaine use (Watkins et al., 2016), while the other found an association between higher spirituality and higher lifetime cocaine use (Allen & Lo, 2010). Spirituality may act as a risk factor for cocaine use in the general population due to aspects such as self-actualization and individualism, which are inherent to some types of spirituality (Ellingson, 2001; Miller, 1998). These pathways potentially connect spirituality to substance use. Sussman et al. (2006) found that there are some aspects of spirituality that act as protective factors for drug use, while others act as risk factors. Therefore, there exists a “non-drug use–specific spirituality” and a “drug use–specific spirituality,” and an individual may tend more toward one or the other based on which factors are most internalized.

Although the overall prevalence of cocaine use has declined slightly in recent years in the USA, it remains a major problem, especially in certain population groups. Among high school students, lifetime cocaine use rose between 2009 and 2015 (Schneider et al., 2018). Emerging adults are also affected, as the prevalence of cocaine use among 18–25-year-olds in 2018–2019 (5.53%) was significantly higher than the prevalence in 2010–2011 (4.68%) (Mustaquim et al., 2021). In this review, five out of six studies conducted on high school students in the USA, with nationally representative data, showed an association between lower cocaine use and higher religiosity (Brown et al., 2001; Dunn, 2005; Fletcher et al., 2014; Palamar & Ompad, 2014; Wallace & Bachman, 1991; Wray-Lake et al., 2012). Additionally, the study conducted on emerging adults (Palamar et al., 2013) demonstrated similar results. Another study conducted in Brazil among emerging adults, excluded from this review because it did not specify the measure of religiosity, showed analogous results (Narvaez et al., 2015).

In the USA, being of Hispanic ethnicity is significantly associated with a higher prevalence of cocaine use and higher cocaine-involved overdose mortality rates than the general population, according to data updated to 2019 and 2018, respectively (Cano, 2021; Schneider et al., 2018). In our review, only one study focused on a sample of people of Hispanic ethnicity (Lamb et al., 2019), which found a direct association between high religiosity and cocaine use. However, this sample also consisted only of gay and bisexual men, and being part of several minorities simultaneously may act as an effect modifier of the association between religiosity and cocaine use. Because our review lacked studies focused on people of Hispanic ethnicity who did not belong to a sexual minority, no further hypotheses could be formulated, and neither could possible repercussions on structuring educational interventions aimed at this group of people be explored.

Indeed, the role of sexual behavior as a modifier effect in the association between religiosity and cocaine use has not been thoroughly investigated. Studies focusing solely on sexual behaviors were conducted only on samples of men belonging to sexual minorities (Lamb et al., 2019; Watkins et al., 2016), in which evidence emerged that increased religiosity was associated with increased cocaine use. Being part of a sexual minority has been associated with a higher risk of substance use initiation (Rosner et al., 2021; Watson et al., 2018), so it is important to understand if religiosity is experienced problematically by sexual minorities, potentially diminishing its protective power against cocaine use.

The two studies we investigated (Lamb et al., 2019; Watkins et al., 2016) hypothesized that the dissonance between sexual orientation and the religious message regarding this aspect may modify the effect. Understanding the specific aspects that undermine the protective power of religiosity on substance use in sexual minorities is crucial for studying targeted interventions tailored to the problems experienced by these individuals. Spirituality may meet the need for a sense of connection with a higher power in people who belong to a sexual minority, without incurring cognitive dissonance. This consideration may explain the results of the study by Watkins et al. (2016).

Although only three studies were conducted outside the USA (Engs & Mullen, 1999; Gmel et al., 2013; Zanetti et al., 2019), focusing on selected samples of university students in Brazil and Scotland and young men about to begin military service in Switzerland, it is noteworthy that the results are generally consistent with those from studies conducted on samples of the general population in the USA. Specifically, two studies, Zanetti et al. (2019) and Gmel et al. (2013), demonstrated that higher religiosity was associated with lower cocaine use among Brazilian university students and young men undergoing military service in Switzerland, respectively.

A significant portion of the studies included in this cohort seems to support the hypothesis that religiosity could be negatively associated with cocaine abuse. These results may be explained by various factors, such as belief in God and a desire to lead a life fully aligned with the teachings and messages of one’s religion. Regarding the protective effect of religiosity against cocaine use, several factors come into play, which can be grouped into two categories (Salas-Wright et al., 2017): aspects related to public religiosity (e.g., frequency of attending religious services, participating in activities within one’s religious community, establishing and maintaining relationships with other members of the religious community) and those related to private religiosity (e.g., importance of religious beliefs, influence on decision-making, degree of adherence to religious teachings). In our review, only two studies (Fothergill et al., 2009; Grunbaum et al., 2000) focused solely on aspects related to public religiosity to assess exposure, specifically the frequency of attending religious services. Both studies demonstrated an inverse association between this aspect and cocaine use. Additionally, four out of five studies (Degenhardt et al., 2007; Dunn, 2005; Engs & Mullen, 1999; Nicholson & Ford, 2019; Zanetti et al., 2019) that exclusively considered aspects related to private religiosity found a protective effect of private religiosity on cocaine use.

Among the studies in our review that analyzed both private religiosity and public religiosity (Salas-Wright et al., 2012, 2015) in adolescents and emerging adults, it was found that while private religiosity alone may not function as a protective factor, the combination of private religiosity with moderate to high public religious participation effectively buffers young people from involvement in cocaine use. These findings support the evidence that both private religiosity and public religiosity, in different ways, play a protective role in cocaine use. Public religiosity may help individuals establish a supportive social network, and peer influence also plays a significant role in preventing cocaine use (Salas-Wright et al., 2012). On the other hand, private religiosity may play a crucial role in moderating the behavior of the faithful. The more central religious teachings are considered in the decision-making process of individuals’ lives, the more they will strive to adhere to precepts that condemn the use of substances (Nicholson & Ford, 2019). Incorporating elements related to religiosity into educational interventions aimed at dissuading cocaine use presents an opportunity to consider, especially in population groups most prone to cocaine use disorder, such as adolescents and young adults. Religiosity has been observed to play a protective role in these groups. Projects adapted to the specific characteristics of youth and implemented in contexts frequented by young people, such as schools, universities, and youth recreational clubs, could be effective.

### Strength and Limitations

This study represents the first systematic review, to our knowledge, of the evidence regarding the association between individuals’ religiosity/spirituality (R/S) and their cocaine use.

However, our systematic review has several limitations. Firstly, the studies included in our review utilized different tools to measure religiosity and cocaine use, with only two studies employing validated questionnaires for assessing religiosity. This diversity in measurement tools prevented us from conducting a meta-analysis. Moreover, only two studies measured spirituality, further limiting our analysis. Standardizing measurement tools in future studies would facilitate comparisons across research efforts. Moreover, it would be desirable that future research should attempt to distinguish the differences between "religiosity" and "spirituality" with regard to cocaine use.

Secondly, the majority of studies were conducted in the US, with only three studies conducted outside the USA focusing on specific populations such as university students or young men about to begin military service. This limits the generalizability of our findings to broader populations.

Thirdly, some of the samples included in our review were drawn from vulnerable populations, such as ethnic or sexual minorities. The characteristics of these populations could influence the association between the variables analyzed, but our review did not evaluate potential modifier effects due to the restricted focus on minority groups. Future studies should explore the influence of different socio-demographic characteristics, such as sexual orientation, on mediating the association between R/S and cocaine use.

Fourthly, the majority of studies included in our review were cross-sectional, limiting our ability to establish temporality and causality. Only two longitudinal studies were available, with one supporting the hypothesis of a proactive role of religiosity and the other showing mixed results. Further longitudinal studies are warranted to address temporality and causality issues.

However, due to the nature of the exposure variable (religiosity), more robust designs such as randomized controlled trials may not be feasible. Observational studies remain crucial for identifying which components of religiosity play a protective role in discouraging cocaine use. Consequently, the results of these observational studies are essential for informing the development of interventions tailored to different settings and population groups.

## Conclusion

This systematic review on the association between religiosity/spirituality (R/S) and cocaine use revealed evidence of a connection between higher levels of religiosity and a decreased likelihood of cocaine use, both in terms of lifetime and current usage. This association was observed across various age groups, including adults and adolescents. However, to validate these findings, further longitudinal studies conducted on representative populations are required. Given these insights, there are promising opportunities for preventive interventions aimed at discouraging cocaine use by incorporating elements related to Religiosity. These interventions could be implemented in diverse settings, tailored to address the specific characteristics and needs of groups most affected by cocaine use, such as young people, sexual minorities, and ethnic minorities. By integrating aspects of Religiosity into preventive environments, we may create contexts that effectively deter cocaine use among vulnerable populations (Tables 2, 3, 4, 5 and 6).

## Data Availability

Not applicable.

## Appendix 1

See Tables

**Table 2:** Methodological appropriateness of the studies, STROBE check list

STROBE check list	1a	1b	2	3	4	5	6a	6b	7	8	9	10	11	12a	12b	12c	12d	12e	Total Score
Palamar et al. (2013) Addiction Research and Theory USA	1	1	1	1	1	1	1	n.a	1	1	1	1	1	1	1	1	1	1	29
Gmel et al. (2013) Subst Use Misuse. Switzerland	0	1	1	1	1	1	1	n.a	1	1	1	1	1	1	1	1	1	1	29
Fletcher et al. (2014) Journal of Economic Behavior and Organization USA	1	1	1	1	1	1	1	0	1	1	0	1	1	1	0	1	1	1	27
Degenhardt et al. (2007) Drug Alcohol Depend. USA	0	0	1	1	1	1	1	n.a	1	1	0	1	1	1	1	0	1	1	26
Watkins et al. (2016) J Relig Health USA	0	1	1	1	1	1	1	n.a	1	1	0	1	1	1	1	0	1	1	26
Salas.Wright et al. (2012) J Youth Adolesc USA	0	1	1	1	1	1	1	n.a	1	1	0	1	1	1	1	0	1	1	25
Zanetti et al. (2019) Texto e Contexto Enfermagem Brazil	1	1	1	1	1	1	1	n.a	1	1	0	1	1	1	1	0	1	1	25
Wray-Lake et al. (2012) J Adolesc Health USA	1	1	1	1	1	1	1	n.a	1	1	0	1	1	1	0	0	1	1	25
Wallace et al. (1991) Social Problems USA	0	1	1	1	1	0	1	n.a	1	1	0	1	1	1	1	0	1	1	25
Salas-Wright et al. (2015) Emerging Adulthood USA	0	1	1	1	1	1	1	n.a	1	1	1	1	1	1	0	0	1	1	24
Brown et al. (2001) Prev Sci USA	0	1	1	1	1	1	1	n.a	1	1	0	1	1	1	1	1	1	1	24
Lamb et al. (2019) Addict Behav. USA	0	1	1	1	1	0	1	n.a	1	1	0	1	1	1	1	0	1	1	24
Nicholson et al. (2019) Addict Behav. USA	0	1	1	1	1	1	1	n.a	1	1	0	1	1	1	0	0	1	1	24
Palamar et al. (2014) Am J Drug Alcohol Abuse USA	0	1	1	1	1	1	1	n.a	1	1	0	1	1	1	1	0	1	1	24
Engs et al. (1999) Addiction Research and Theory Scotland	0	1	1	1	1	1	1	n.a	1	1	0	1	1	1	1	0	1	1	23
Grunbaum et al. (2000) Addict Behav. USA	0	1	0	1	1	1	1	n.a	1	1	0	1	1	1	1	0	1	1	23
Miller et al. (2000) J Am Acad Child Adolesc Psychiatry USA	0	1	1	1	1	1	1	n.a	1	1	0	1	1	1	1	0	1	1	23
Fothergill et al. (2009) J Health Soc Behav. USA	0	1	1	1	1	1	0	0	1	1	0	1	1	1	0	0	1	1	22
Allen et al. (2010) Journal of Drug Issues USA	0	1	1	1	1	1	1	n.a	1	1	0	1	1	1	0	0	1	1	21
Dunn (2005) Journal of Alcohol and Drug Education USA	0	1	1	1	1	1	1	n.a	1	1	0	1	1	1	1	0	1	1	20

STROBE check list	13a	13b	13c	14a	14b	14c	15	16a	16b	16c	17	18	19	20	21	22	Total Score
Palamar et al. (2013) Addiction Research and Theory USA	1	1	0	1	0	n.a	1	1	1	1	1	1	1	1	1	0	29
Gmel et al. (2013) Subst Use Misuse. Switzerland	1	1	0	1	0	n.a	1	1	1	1	1	1	1	1	1	1	29
Fletcher et al. (2014) Journal of Economic Behavior and Organization USA	1	0	0	1	0	1	1	1	1	1	1	1	1	1	1	0	27
Degenhardt et al. (2007) Drug Alcohol Depend. USA	1	1	0	1	0	n.a	1	1	1	1	1	1	1	1	1	1	26
Watkins et al. (2016) J Relig Health USA	1	0	0	1	1	n.a	1	1	1	1	1	1	1	1	1	0	26
Salas.Wright et al. (2012) J Youth Adolesc USA	1	0	0	1	0	n.a	1	1	1	1	1	1	1	1	1	0	25
Zanetti et al. (2019) Texto e Contexto Enfermagem Brazil	1	0	0	1	0	n.a	1	1	1	1	1	1	1	1	0	0	25
Wray-Lake et al. (2012) J Adolesc Health USA	1	0	0	1	0	n.a	1	0	1	1	1	1	1	1	1	1	25
Wallace et al. (1991) Social Problems USA	1	1	0	1	0	n.a	1	1	1	1	1	1	1	1	1	0	25
Salas-Wright et al. (2015) Emerging Adulthood USA	1	0	0	1	0	n.a	1	1	0	1	1	1	1	1	1	0	24
Brown et al. (2001) Prev Sci USA	1	1	0	0	0	n.a	0	0	1	1	1	1	1	1	1	0	24
Lamb et al. (2019) Addict Behav. USA	1	1	0	1	0	n.a	1	1	1	1	0	1	1	1	1	0	24
Nicholson et al. (2019) Addict Behav. USA	1	1	0	1	0	n.a	1	1	1	1	0	1	1	1	1	0	24
Palamar et al. (2014) Am J Drug Alcohol Abuse USA	1	0	0	1	0	n.a	1	1	1	1	1	1	1	1	0	0	24
Engs et al. (1999) Addiction Research and Theory Scotland	1	1	0	0	0	n.a	1	1	0	1	1	1	0	1	1	0	23
Grunbaum et al. (2000) Addict Behav. USA	1	1	0	1	0	n.a	1	1	0	1	1	1	0	1	1	0	23
Miller et al. (2000) J Am Acad Child Adolesc Psychiatry USA	1	1	0	0	0	n.a	0	1	1	1	0	1	1	1	1	0	23
Fothergill et al. (2009) J Health Soc Behav. USA	1	0	0	1	0	1	1	0	1	1	1	1	1	1	0	0	22
Allen et al. (2010) Journal of Drug Issues USA	1	0	0	1	0	n.a	1	0	0	1	0	1	1	1	1	0	21
Dunn (2005) Journal of Alcohol and Drug Education USA	1	0	0	0	0	n.a	1	0	1	1	0	1	0	1	0	0	20

^2^,

**Table 3:** Overview of studies measuring only religiosity reviewed. Material and methods

Author Year Journal	Adolescent (AD) Adult (A)	Study design Sample methods	Sample Age	Measure of exposure	Measure of Outcome
Dunn, 2005 Journal of Alcohol and Drug Education	AD and A	Cross-sectional study Data from the Monitoring the Future (MTF) study (2002 12th-grade core data) were used. The sampling procedure for the MTF is a multistage probability sample design based on geographical area or primary sampling units (PSUs), schools within PSUs, and students within the sampled school	USA, Year 2002 6029 participants, both < 18 and 18 + . Inclusion criteria: being high school students	**Type of religion**: not specified **Measure**: based on the question “ how important is religion in your life?” the measure was tri-chotomized into little important, pretty important, and very important	A single question: “ On how many occasions (if any) have you taken cocaine in your lifetime?” the outcome was dichotomized into yes/no categories
Engs & Mullen, 1999 Addiction Research and Theory	A	Cross-sectional study The sample was a convenience sample collected during autumn term 1994 at Scottish tertiary institutions; 22 departments of medicine, nursing, education, psychology, and social work asked their students to participate	Scotland, Year 1994 4066 participants, students in the helping professions of Scottish tertiary institution	**Type of religion**: Church of Scotland, Church of England, Other Protestant, Roman Catholic, Other or None **Measure**: based on the question “how important religion was to them (“very,” “moderately,” “slightly,” “not at all”), then dichotomized in “Very Religious” and “Not Religious”	The Queensland Alcohol and Drug Study Questionnaire was used. Cocaine use was assessed with the question “how frequently have you used cocaine over the past year?” Students were categorized as “users” versus “non-users”
Fletcher et al. 2014 Journal of Economic Behavior and Organization	AD and A	Longitudinal study In this study was used the restricted version of the National Longitudinal Study of Adolescent Health (Add Health), a nationally representative study of 7–12th-grade students, their parents (or guardians), and school administration. The Add Health sample follows a stratified sampling design based on region, urbanization, school type, ethnic mix, and size. In this study, data from Wave 1 (1994–1995), Wave 3 (2001–2002) and Wave 4 (2007–2008) were used, plus the siblings of the surveyed students	USA, Year 1994–1995, 2001–2002, 2007–2008 Around 15.000 participants. Inclusion criteria: being 7-12th-grade students in wave 1 (1994–1995)	**Type of religion**: Other religions, no religion, Catholic, Moderate Protestant, Liberal Protestant, Conservative Protestant, Jewish **Measure:** two measures of extrinsic religiosity come from the following questions: (1) for religious attendance: “In the past 12 months, how often did you attend religious services?” Answers categories ranged from no religion, never, less than once a month, once a month or more/less than once a week, to once a week or more; (2) for prayer frequency: “How often do you pray?” the categories were no religion, never, less than once a month, at least once a month, to at least once a week. The measure of intrinsic religiosity comes from the question, “How important is religion to you?” Answers categories ranged from no religion, not important at all, fairly unimportant, fairly important, to very important. Aa value of 0 was assigned to those who report no religion or do not value religious rites and rituals	A single question for each type of substance: “Since June 1995, have you used any kind of cocaine—including crack, freebase, or powder?”; for methamphetamine: “Since June 1995, have you used crystal meth?”; for the measure of other drugs use: “Since June 1995, have you used any other types of illegal drug, such as LSD, PCP, ecstasy, mushrooms, inhalants, ice, heroin, or prescription medicines not prescribed for you?” The outcome was dichotomized into yes/no categories
Palamar et al., 2013 Addiction Research and Theory	A	Cross-sectional study Targeted sampling strategies were applied to obtain a purposive sample of emerging adults. Recruitment was conducted throughout Manhattan, New York, in parks, at college campuses, and near other locations frequented by emerging adults. Respondents were recruited on the street. One out of every three individuals or groups who appeared to be emerging adults was approached	New York, USA, Year 2009 1048 participants. Inclusion criteria: > 18 and < 25, fluent in English, with internet access if they participated via internet	**Type of religion**: not specified **Measure**: Level of religiosity was assessed through two items, which were calculated into a composite score. Religious salience was measured though a four-point Likert scale which asked, ‘‘How important is religion in your life?’’ Answer options ranged from (1) ‘‘Not important’’ to (4) ‘‘Very important.’Level of religious service attendance was also assessed: ‘‘How often have you attended religious services within the last year?’’ Answer options ranged from (1) ‘‘Never’’ to (4) ‘‘About once a week or more.’’	The survey assessed lifetime use (‘‘Have you ever used___?’’) of five drugs: marijuana, powder cocaine, ecstasy, and non-medical use of select amphetamine and opioid prescription drugs. Answer options were ‘‘Yes’’ and ‘‘No.’’
Salas-Wright et al., 2015 Emerging Adulthood	A	Cross-sectional study Data for this study are based on the 2010 NSDUH (Substance Abuse and Mental Health Services Administration [SAMHSA], 2011) and the 2004–2005 NESARC (Grant et al., 2003). Both studies used multistage sampling designs to provide population estimates of substance use and health-related behaviors for the non-institutionalized, civilian population in the USA. This study restricted analyses to emerging adults in the NSDUH and NESARC	USA, Years 2004/2005 for NESARC data, 2010 for NSDUH data. 2721 participants in NESARC, 19.312 participants in NSDUH. Inclusion criteria: age > 18 and < 25, non-institutionalized, civilian	**Type of religion**: not specified **Measure**: In the NSDUH, frequency of religious service attendance was measured by asking respondents: ‘‘During the past 12 months, how many times did you attend religious services?’’ Respondents were categorized into six ordinal groups ranging from no religious service attendance to attendance at more than 52 religious services in the previous year. The remaining 3 items measured the importance and influence of religious beliefs. These items include the following: ‘‘Your religious beliefs are a very important part of your life;’’ ‘‘Your religious beliefs influence how you make decisions in your life;’’ and ‘‘It is important that your friends share your religious beliefs.’’ All three of these items had the response format of strongly disagree, disagree, agree, and strongly agree. In the NESARC, frequency of religious service attendance was measured by asking respondents: ‘‘How often do you currently attend religious services at a place of worship?’’ Response options ranged from do not attend to twice a week or more. Religious social engagement was measured by asking participants, ‘‘How many members of your religious group do you see or talk to socially every two weeks?’’ Response options included ‘‘0,’’ ‘‘1–2,’’ ‘‘3–5,’’ ‘‘6–10,’’ and ‘‘11 or more.’’ Importance of religious beliefs was measured by asking participants, ‘‘How important are religious or spiritual beliefs in your daily life?’’ This item had the response format of very important, somewhat important, not very important, and not important at all	Use of cocaine/crack over the previous 12 months (0 = no, 1 = yes)
Zanetti et al., 2019 Texto e Contexto Enfermagem	A	Cross-sectional study The target population was the students of the courses of Information Sciences and Documentation, Law, Nursing and Occupational Therapy of the campus of a public university of Ribeirão Preto-SP. The convenience sample consisted of 275 students	Brazil, Year 2014 275 participants. Inclusion criteria: > 18, regularly enrolled and perpetuating the referred courses during the year 2014 at public university of Ribeirão Preto	**Type of religion**: not specified **Measure**: a question about the importance attributed to religion, dichotomized in two categories: “very important” and “little important”	Two questions: 1) lifetime use cocaine 2) consumption of cocaine in the last three months. Answers were dichotomized in Yes or No
Brown et al., 2001 Prev Sci	AD and A	Cross-sectional study. Data were drawn from the Monitoring the Future (MTF) project, an ongoing study of young people. Every year since 1975, a multistage, clustered sample of high schools was drawn. Approximately 135 high schools were randomly sampled from the coterminous 48 states, and between 15,000 and 19,000 high school seniors were surveyed each year	USA, Year 1976–1997 188.682 participants. Inclusion criteria: being high school seniors, reporting their race as Black or African American, White, or Hispanic	**Type of religion**: not specified **Measure**: religiosity; average of how often student attends religious services and how important religion is in the student’s life. 1 = very low, 2 = low, 3 = high, 4 = very high	Frequency of cocaine use was assessed by past 12-month cocaine use, with 1 = 0 occasions, 2 = 1–2 occasions, 3 = 3–5 occasions, 4 = 6–9 occasions, 5 = 10–19 occasions, 6 = 20–39 occasions, 7 = 40 or more
Degenhardt et al., 2007 Drug Alcohol Depend	A	Cross-sectional study NCS-R is a nationally representative household survey in the coterminous United States; respondents were drawn by probability sampling within a multistage clustered area probability sample of households; one randomly selected person from each household was sampled. Standardized assessments were completed via computer-assisted personal interviews. The survey was administered in two parts, only part II respondents were evaluated	USA, Year 2001–2003 5692 participants. Inclusion criteria: > 18, English speaking	**Type of religion**: Black Protestant, Evangelical Protestant, Catholic, Jewish, Others, None, Mainline Protestant **Measure**: Religiosity was assessed for all part II respondents, who reported how important religious beliefs were in their lives (low, a little, somewhat, very much)	One question: “Have you ever used cocaine in any form, including powder, crack, free base, coca leaves, or paste?” Answers were dichotomized in Yes or No
Gmel et al., 2013 Subst Use Misuse	A	Cross-sectional study Enrollment in the study took place in three army recruitment centers located in Lausanne, Windisch, and Mels. Of the 13,245 conscripts informed about the study (87.9%), 7,563 (57.1%) gave written consent to participate and 5,990 of those (79.2%) completed the baseline questionnaire. The final sample consisted of 5,387 participants	Switzerland, Year 2010–2011 5387 participants. Inclusion criteria: > 18, males who visited the recruitment centers, giving written consent to participate to the study. Exclusion criteria: not reporting the religious denomination in the questionnaire, having incomplete data on any of the variables, having reported to be Jews, Muslims or “other religion” (because of insufficient sample size of these religions)	**Type of religion**: Protestant, Catholic, Other Christian, no religion **Measure**: Religious denomination -RD- was assessed with the question “What is your religion (even if you do not practice or believe in God)?” The nine possible categories were Roman Catholic, Protestant, Christian-Catholic, Christian Orthodox, Other Christian (the latter three were combined as Other Christian), Jewish, Muslim, Other (individuals from the three latter categories were then excluded from the study), and No RD. Religiosity—measured as RSD, Religious self-description -was measured with the first question on the Religious Background and Behavior Questionnaire. Participants were asked to indicate which of the following five categories described them best: Religious (“I believe in God and practice religion”), Spiritual (“I believe in God but do not practice religion”), Unsure (“I do not know what to believe about God”), Agnostic (“I believe we cannot really know about God”), or Atheist (“I do not believe in God”)	One question about the use of cocaine during the past 12 months. Answers were dichotomized in Yes or No
Grunbaum et al., 2000 Addict Behav	AD and A	Cross-sectional study Fifty-one schools within Texas were identified as dropout prevention and recovery high schools. Of the 51 schools, 16 had at least 100 students enrolled and were invited to participate in the study, and five schools agreed to participate. The enrollment across the five schools was 543 students; of those, 475 completed the survey (85%). 31 students were then excluded, leaving a total of 441 subjects	Texas, USA, Year 1997 441 participants. Inclusion criteria: being high school students. Exclusion criteria: having recording ethnicity as “other”	**Type of religion**: not specified **Measure**: religiosity was assessed with the question “During the past year, how often have you attended religious services?”; answers were dichotomized in “at least once monthly” and “infrequently (less than once monthly) or never”	Substance use in the past month, including use of cigarettes, alcohol, marijuana, and cocaine, was assessed using questions from the Youth Risk Behavior Survey (YRB). Each substance was coded 0 (no use in past month) or 1 (used at least once in past month)
Lamb et al., 2019 Addict Behav	A	Cross-sectional study Participants were recruited through paid advertisements on several hook-up sites, such as Grindr	San Diego County, USA, Year of data collection not specified 151 participants. Inclusion criteria: > 18 and < 29, males, identifying as a gay/bisexual male or reported same sex attraction, Latino/Hispanic ethnicity, self-reporting HIV-uninfected status, Spanish or English speaking, living in San Diego County	**Type of religio**n: not specified **Measure**: Religiosity was assessed using the three subscales of the Duke University Religion Index: organizational religious activity (ORA), non-organizational religious activity (NORA), and intrinsic religiosity (IR). ORA was assessed with the item: “How often do you attend church or other religious meetings?” Responses ranged from 1 = Never to 6 = More than once/ week. NORA was assessed with the item: “How often do you spend time in private religious activities, such as prayer, meditation or Bible study?” Responses ranged from 1 = Rarely or never to 6 = More than once a day. IR was assessed using the three items: “In my life, I experience the presence of the Divine (i.e., God),” “My religious beliefs are what really lie behind my whole approach to life,” and “I try hard to carry my religion over into all other dealings in life.” Responses were measured on a 5-Point Likert scale (e.g., 1 = Definitely not true and 5 = Definitely true to me). The three IR items were summed to create a scale score, possible scores ranged from 3 to 15	Cocaine use was assessed with the following item—“Please select the answer that most accurately describes your cocaine use in the past month. Responses ranging from 1 = No use to 5 = About every day. Dichotomous variables were created: 0 = no cocaine use vs. 1 = cocaine use in the past month
Miller et al., 2000 J Am Acad Child Adolesc Psychiatry	AD	Cross-sectional study The study used data from adolescents in the National Comorbidity Survey (NCS), a nationally representative household survey designed to study patterns and correlates of psychiatric morbidity and comorbidity	USA, Years 1990–1992 676 participants. Inclusion criteria: > 15 and < 18, having responded to all 7 of the religiosity items	**Type of religion**: fundamentalist protestant, baptist, mainline protestant, roman catholic, other or unaffiliated **Measure**: Religiosity was assessed by using responses to 7 items 1) How important are religious or spiritual beliefs in your daily life? 2) How often do you attend religious services? 3) When you have problems in your life how often do you seek spiritual comfort? 4) When you have decisions to make in your daily life, how often do you ask yourself what God would want you to do? 5) Have you been “born again” that is had a turning point in your life when you commit yourself to Jesus? 6) Do you encourage people to believe in Jesus and accept Him as their Savior? 7) The Bible is the actual Word of G-d and is to be taken literally, word for word. (extent agree or disagree). From the answers, personal devotion and institutional conservatism scores were assessed	Cocaine use was assessed on the basis of the following questions: ever used cocaine; age of first use of cocaine; frequency of use of cocaine during the past year. Diagnosis of substance dependence and substance abuse, as a single category, was based on DSM-111-R criteria
Nicholson & Ford, 2019 Addict Behav	A	Cross-sectional study The study used data from the NSDUH, a nationally representative sample of non-institutionalized persons aged 12 and older in the USA. After pooling two years of data (2015, 2016), the full sample included 114,043 respondents. Data were pooled across two years to generate a larger sample size of Blacks. The final analytical sample consisted of 77,930 adults, including 9821 non-Hispanic Blacks	USA, Years 2015–2016 9821 participants. Inclusion criteria: > 18, being Blacks	**Type of religion**: non-specified **Measure**: religiosity was assessed with the question “Your religious beliefs influence how you make decisions in your life?”; possible answers were dichotomized agree/ strongly agree or disagree/strongly disagree	Cocaine use was assessed with the self-reported past-year use of powder cocaine, “crack,” free base, or coca paste cocaine. Answers were dichotomized in Yes or No
Palamar & Ompad, 2014 Am J Drug Alcohol Abuse	AD and A	Cross-sectional study MTF is an annual survey of high school seniors in approximately 130 public and private schools throughout 48 states in the USA. Schools are selected through a multistage random sampling procedure: geographic areas are selected, then schools within areas are selected, and then, students within schools are selected. This analysis focuses on aggregated (and weighted) data from 65 717 high school seniors from years 2005–2011	USA, Year 2005–2011 65,717 participants. Inclusion criteria: being high school seniors	**Type of religion**: not specified **Measure**: Level of religiosity was determined by two ordinal items addressing religious attendance and importance. These items were computed into a mean religiosity composite (range: 1–4) and divided into tertiles indicating low (1.0–2.0), moderate (2.5–3.0), and high (3.5–4.0) religiosity	Lifetime use of cocaine powder or crack, answers were dichotomized in Yes or No
Salas. Wright et al. (2012) J Youth Adolesc	AD	Cross-sectional study This study is based on public-use data from the 2008 National Survey on Drug Use and Health (NSDUH). It utilizes multistage area probability sampling methods to select a representative sample of the US civilian, non-institutionalized population aged 12 years or older for participation in the study. All 50 states and the District of Columbia were employed. To improve the precision of drug use estimates for subgroups, adolescents aged 12–17 years and young adults aged 18–25 years were oversampled	USA, Year 2008 17,705 participants. Inclusion criteria: > 12 and < 17, non-institutionalized	**Type of religion**: not specified **Measure**: Five items were used to assess various forms of religiosity and religious participation; Frequency of religious service attendance was measured by asking respondents: ‘‘During the past 12 months, how many times did you attend religious services (excluding special occasions such as weddings, funerals, etc.).’’ Youth were categorized into five ordinal groups ranging from no religious service attendance to attendance at more than 52 religious services in the previous year. Participation in religious groups was measured by asking respondents: ‘‘During the past 12 months, in how many different kinds of church or faith-based activities, such as clubs, youth groups, Saturday or Sunday school, prayer groups, youth trips, service or volunteer activities have you participated?’’ Youth were categorized into four ordinal categories ranging from no groups to three or more groups. Importance of religious beliefs was measured by asking respondents the degree to which they agreed with the following statement: ‘‘Your religious beliefs are a very important part of your life.’’ Religious influence was measured by asking respondents the degree to which they agreed with the following statement: ‘‘Your religious beliefs influence how you make decisions in your life.’’ Finally, importance of shared peer beliefs was measured by asking respondents the degree to which they agreed with the following statement: ‘‘It is important that your friends share your religious beliefs.’’ The last three of these items had the response format of strongly disagree, dis agree, agree, and strongly agree	Use in the previous 12 months of cocaine/crack was assessed. Use of either powder cocaine or crack was coded as 1 while youth who reported no use of either cocaine/ crack was coded as 0
Wray-Lake et al., 2012 J Adolesc Health	A and AD	Cross-sectional study Were used 33 survey years (1976 to 2008) of data from Monitoring the Future, an ongoing nationally representative study of high school seniors in the USA. The study draws samples of the same age group (high school seniors, modal age 18) from different cohorts (successive graduating classes) at different times (each year from 1976 to 2008). Each year, 12th-grade students were selected using a multistage random sampling procedure of public and private high schools nationwide	USA, Year 1976–2008 64,246 participants, both > 18 and < 18. Inclusion criteria: being 12th-grade students in high schools	**Type of religion**: not specified **Measure**: One item measured frequency of attending religious services on a 4-point scale: never (1), rarely (2), once or twice a month (3), and about once a week or more (4). A second item asked how important religion was in their lives using a 4-point scale: not important (1), a little important (2), pretty important (3), and very important (4). The items were then combined in a unique dimension	Lifetime and past 30-day use of cocaine or crack were computed by calculating any (1) versus no use (0). Only for lifetime use OR has been calculated
Wallace & Bachman, 1991 Social Problems	AD and A	Cross-sectional study The data used in this study are drawn from the Monitoring the Future project, which involves large, nationally representative samples of high school senior. The study uses a multistage sampling procedure, which results in samples representative of high school seniors in the 48 coterminous states. First, particular geographic areas are selected. Next, schools are selected—approximately 135 schools participate each year. Finally, up to 400 seniors are selected in each school, by randomly selecting classrooms or some other unbiased method	USA, Year 1985–1989 77,500 participants, both > 18 and < 18. Inclusion criteria: being high school seniors	**Type of religion**: not specified **Measure**: religiosity was a mean of how often student attends religious services and how important religion is in the student’s life. 1 = very low, 2 = low, 3 = high	One item: on how many occasions respondents used cocaine (sometimes called “coke,” “crack,” or “rock”), during the last 12 months; 1 = 0 occasions, 2 = 1–2, 3 = 3–5, 4 = 6–9, 5 = 10–19, 6 = 20–39, 7 = 40 or more
Fothergill et al., 2009 J Health Soc Behav	A	Longitudinal study A cohort of males and females (who began first grade in 1966–67 in Woodlawn, an inner-city community on the south side of Chicago) has been followed for more than 35 years. Participants were adults when answered about cocaine use	USA, Chicago, Years 1966–1967,1975–1976, 1992–1994, 2002–2003 1,242 participants, males and females. Inclusion criteria: began first grade in 1966–67 in Woodlawn	**Type of religion**: not specified **Measure**: self-reports of frequency of church attendance. Responses ranged from 1 = less than once a year to 6 = several times per week. The same variable was included in mid adulthood to control for current church attendance	Self-reports of frequency and recency f cocaine use in the past 10 years. Frequency responses ranged from 0 = none in the past 10 years to 7 = more than 200 times. The recency responses were categorized as 0 = never or not at all in the past 10 years, 1 = in the past 1–10 years, and 2 = within the past year

3,

**Table 4:** Overview of studies measuring both religiosity and spirituality reviewed. Material and methods

Author Year Journal	Adolescent (AD) Adult (A)	Study design Sample methods	Sample Age	Measure of exposure	Measure of Outcome
Allen & Lo, 2010 Journal of Drug Issues	A	Cross-sectional study Data were drawn from the general Social Survey of 2004, conducted by the National Opinion Research Center at the University of Chicago	USA, Year 2004 2812 participants. Inclusion criteria: > = 18, English speaking, non-institutionalized	**Type of Religion**: not specified **Measure**: an index of a respondent’s religiosity was created, summing the standardized scores for three measures: (a) religious fundamentalism (response categories were fundamentalist, moderate, and liberal); (b) frequency of religious attendance (8 response categories, ranging from never to several times weekly); and (c) strength of religion (4 response categories, ranging from no to strong). Also a spirituality index with an ad hoc 11-items questionnaire was created, with questions about frequency of (a) individual prayer, (b) feeling divine love directly, (c) asking for divine help, (d) feeling divine guidance, (e) feeling thankful for blessings, (f) desiring to be closer to the divine, (g) gaining strength from religion/spirituality, (h) gaining comfort from religion/spirituality, (i) feeling connected to all of life, (j) feeling joy lifting one out of daily concerns, and (k) feeling divine love through others (there were 6 response categories, ranging from never to many times a day)	A single question about the lifetime use of crack cocaine (1 for yes, 0 for not)
Watkins et al., 2016 J Relig Health	A	Cross-sectional study The study used a subset of data from the 2005 Brothers y Hermanos Study (ByHS) rigorously designed and assessed a broad range of relevant areas. Data set provides a very large sample (N = 1140) of Black MSM (male sexually active with another male) recruited from large urban areas	USA, Year 2005 1141 participants. Inclusion criteria: > 18, born male, black, sexually active with another male within the past 12 months	**Type of religion**: not specified **Measure**: A religiosity index was created by summing the scale responses of the four religiosity questions from the ByHS data: worship (0–4) + openness (0–4) + religious beliefs (0–4) + choosing religious beliefs (0–4) to develop a composite sum of the responses to the four original ByHS religiosity questions. A spirituality index was created by using participant responses to three questions by summing guidance (0–4) + spiritual connection (0–4) + spirituality and health (0–4)	Lifetime use of cocaine powder or crack, answers were dichotomized in Yes or No

4,

**Table 5:** Overview of studies measuring only religiosity reviewed. Results

Author Year Journal	Results	Confounder	Conclusion
Dunn, 2005 Journal of Alcohol and Drug Education	Males and females who believed religion was important were significantly more likely to have not tried cocaine: OR = 1.33, CI 95% (1.19–1.56) in males; OR = 1.30; CI 95% (1.17–1.53) in females. The referent group for calculating OR was “little importance of religion”	gender, political belief, working status	The results of this study seem to indicate that development of alcohol use patterns, cigarette use, and cocaine use are related to holding liberal views, not thinking religion is important, and working moderate to high amounts after school
Engs & Mullen, 1999 Addiction Research and Theory	In Chi-square analysis, the percent of female students who use cocaine were 1.8 if very religious, 2.4 if not religious; but weren’t statistically significant. The percent of male students were 3.1 if very religious, 4.4 if not religious, but weren’t statistically significant too	gender	Cocaine use was reported by 4.0% of males and 2.2% of females. For both the female and male samples, there was no significant difference due to religiosity (importance of religion) and the use of cocaine. About twice as many individuals who gave Other or No religious preference had used cocaine during the previous 12 months. For females there was a significant difference between religious background (type of religion) (X2 = 9.7, p < .05); there was no significant difference between religious groups among males
Fletcher et al. 2014 Journal of Economic Behavior and Organization	On the full sample, the OLS estimated coefficients (fixed for school effects) were -0.015 for cocaine use and Religious attendance (p < 0.01); -0.08 for cocaine use and Prayer frequency (p < 0.05); and -0.024 for cocaine use and importance of religion (p < 0.01); but when fixed for family effects they all have no longer significance (p > 0.1). In the siblings sample, cocaine use and importance of religion, at individual level (without family fixed effect), were associated with 10% level of significance, with -0.019; all the other estimated coefficients between cocaine use and measures of religiosity, at individual level or at family level, were not statistically significant	religious affiliation (Catholic, Moderate Protestants, etc.), age, gender, ethnicity, household size, family income, parental	
education, parental age, presence of biological mother and father	In the sample of siblings, the OLS estimates (without family fixed effects) suggest that all three measures of religion reduce the propensity to use the illicit drugs in the medium as well as in the longer term; but, except for methamphetamine use in Wave 3 for the intrinsic religiosity measure, none of the estimates are statistically significant. In the full sample, when estimates were fixed for school effects, it shows a protective effect of all three measures of religion toward cocaine use, and the estimates were statistically significant; but when estimates were fixed for family effects, it is no longer significant. Therefore, family was found to be an institution that plays a very significant role in influencing the relationship between religiosity and cocaine use behaviors		
Palamar et al., 2013 Addiction Research and Theory	Results of age-adjusted multivariate logistic regression predicting lifetime powder cocaine use show a OR = 0.66 (*p* < 0.01) CI 95% (0.52–0.83) for religiosity in step 2, and OR = 0.69 (*p* < 0.01) CI 95% (0.55–0.88) in step 3. In the final step, for every unit increase in religiosity, the odds of use decreased 31%	age, gender, education level, ethnicity, exposure to users of substance, perceived public stigma toward users of substance	In the final step of multivariate logistic regression, for every unit increase in religiosity, the odds of use of cocaine decreased 31%. Religiosity is more protective against use of the ‘‘hard drugs,’’ cocaine and ecstasy, but it is not as protective against lifetime use of the other drugs, over and above stigma, and exposure; maybe cocaine and ecstasy might receive higher levels of moral or religious disapproval in addition to general stigma
Salas-Wright et al., 2015 Emerging Adulthood J Health Soc Behav	For both NESARC and NSDUH samples, subjects have been assigned to four different classes of religiosity: Class 1: ‘‘publicly and privately disengaged’’; Class 2: ‘‘low public and private religiosity’’; Class 3: ‘‘moderate public and private religiosity’’ and Class 4: “publicly and privately devoted.” In NSDUH sample, members of class 2 (RR = 0.78, 95%CI 0.61–0.99, *p* < 0.05), class 3 (RR = 0.45, 95%CI 0.35–0.58, *p* < 0.05) and class 4 (RR = 0.17, 95%CI 0.11–0.25, *p* < 0.05) were significantly less likely to use cocaine/crack; the reference was class 1. In NESARC sample, members of class 3 (RR = 0.52, 95%CI 0.45–0.62, *p* < 0.05) and class 4 (RR = 0.05, 95%CI 0.04–0.06, *p* < 0.05) were significantly less likely to use cocaine/crack	age, gender, ethnicity, education level, total annual family income	Membership of the moderate (class 3) and high (class 4) public and private religiosity classes is powerfully linked with the decreased likelihood of meeting criteria for cocaine, nicotine, alcohol, marijuana, and other illicit drug use disorders. There is also a decreased likelihood of involvement in antisocial behavior
Zanetti et al., 2019 Texto e Contexto Enfermagem	There was a significant association between the importance of religion and lifetime use of cocaine (*p* = 0.024) and between the importance of religion and the last three months use of cocaine (*p* = 0.098). A Fisher’s Exact test was use; the significance level was set at 0.05		Regarding religion, the percentage of university students who reported using alcohol, marijuana, and cocaine in their lives or use of marijuana in the last three months is higher among those who do not find the religion important (significant association)
Brown et al., 2001 Prev Sci	Coefficients from OLS regression of annual cocaine use on religious commitments ranged from -0.128 to -0.017 (*p* < 0.001) in nine years surveyed	gender, ethnicity, parental education, urbanicity, region, political beliefs, truancy, college plans, employment, social interaction	Controlling for historical time period, predictors consistently linked to cocaine use were: being Black (Whites higher), number of parents in household (negative), religious commitment (negative), political beliefs (positive), grade point average (negative), truancy (positive), and evenings out (positive). In contrast, parental education, urbanicity, region, college plans, total weekly income, and number of dates per week were inconsistently linked to cocaine use over time
Degenhardt et al., 2007 Drug Alcohol Depend	The low self-reported importance of religion was associated with cocaine use: OR = 1.4, with CI 95% (1.1–1.9), *p*-value < 0.05. Those for whom religion was less important were more likely to have used all drug types, included cocaine	age, sex, tobacco use, alcohol use, cannabis use, extra-medical drug use	The inverse association of importance of religion with cocaine use largely remained after covariate adjustment, but the pattern was more akin to a threshold function, with those who held religion as “very important” to them being less likely than others to have become drug users
Gmel et al., 2013 Subst Use Misuse	Regarding Religious Self-Description, the proportion of cocaine users among those self-describing as “religious” individuals was lower respect all the other categories (religious at 0,7; spiritual at 2,7; unsure at 2,4; agnostic at 3,1; atheist at 5,1). Respect to those defining themselves as “ religious,” adjusted OR for cocaine use and those self-describing as “atheist” was 5.13, CI 95% (1.83–14.40) *p* < 0.01	parental relationship, parental regulation, parental monitoring	Religiosity, measured as RSD, had a stronger impact on substance use than religious denomination (RD). The study confirmed that RD and RSD can operate as independent protective factors for licit and illicit substance use, under conditions to be delineated, and that their effects do not only depend on parental influence in adolescents and young adults
Grunbaum et al., 2000 Addict Behav	Those attending church at least once monthly were significantly less likely to use cocaine as compared to students who infrequently or never attend church. OR for cocaine use in the past month and religion was 0.46 CI 95% (0.24–0.89)	gender, ethnicity, age, family caring, depression, loneliness, educational aspiration	Factors associated with cocaine use in the final multivariate model included being a younger age, family caring, less coping ability, church attendance, and low educational aspirations
Lamb et al., 2019 Addict Behav	NORA (non-organizational religious activity) was significantly associated with increased cocaine use (OR = 1.69, SE = 0.20, CI 95% 1.14–2.52, *p* = .01). ORA (organizational religious activity) was not associated with increased cocaine use (OR = 1.41, SE = 0.19, CI 95% 0.96–2.07, *p* < 0.1). IR (intrinsic religiosity) was not associated with increased cocaine use (OR = 0.93, SE 0.10, CI 95% 0.76–1.13)	ethnicity, age	Results somewhat supported the hypothesis ORA and NORA would be significantly associated with increased illicit substance use. Additionally, the results did not support the hypothesis IR would be significantly associated with decreased use of multiple illicit substances; a potential explanation for IR not being associated with illicit substance in the current sample is that Latino Sexual Minority Men in religious communities may experience stress as a product of the interaction of their sexual orientation with their religious involvement, thereby making the protective effects of religion on health less salient in this population. NORA would be associated with greater substance use in Latino SMM potentially due to the fact greater internalization of negative religious beliefs toward SM individuals can lead to greater stress and negative health outcomes
Miller et al., 2000 J Am Acad Child Adolesc Psychiatry	In the regression analyses, an increase in personal devotion or institutional conservatism were found to be associated with decreased likelihood of having ever used cocaine. Adjusted OR = 0.50 (CI 95% 0.31–0.79, *p* < 0.01) for lifetime use of cocaine and personal devotion, adjusted OR = 0.58 (CI 95% 0.38–0.90, *p* < 0.05) for lifetime use of cocaine and institutional conservatism	age, gender	Personal devotion and institutional conservatism were strongly inversely associated with the use of a range of substances (alcohol, marijuana, cocaine, or any contraband drug) and strongly inversely associated with substance dependence or abuse
Nicholson & Ford, 2019 Addict Behav	Religiosity was associated with decreased odds of Cocaine use (OR = 0.55, CI 95% 0.32–0.92, *p* < 0.05)	gender, age, income, education level, marital status, employment status, geographical location, depression, overall health	Results matched past research on the effects of religiosity on Cocaine use among Blacks. In addition to treatment, incorporating religious elements into prevention and intervention programs may be effective for some Blacks at risk of and currently using cocaine
Palamar & Ompad, 2014 Am J Drug Alcohol Abuse	Higher religiosity robustly and consistently lowered the odds of use of both crack and powder cocaine, with, respectively, OR = 0.45, CI 99% 0.35–0.57, *p* < 0.0001 and OR = 0.29, CI 99% 0.24–0.35, *p* < 0.0001	age, gender, ethnicity, parent education, family structure, weekly income	Religiosity lowered odds for use of both powder and crack cocaine, consistent with previous research demonstrating that higher religiosity tends to be protective against use of most illicit drugs. However, when examining differences between types of cocaine users, cocaine-only users tended to be less religious, and crack users (with or without use of powder cocaine) tended to be more religious. This association was most consistent in crack only users. It is possible that religious individuals were more likely to use crack, but it is also possible that that crack users were more likely to become religious after use
Salas et al. (2012) J Youth Adolesc	A series of latent profile models from 1 to 5 classes were carried out in order to identify distinct profiles of adolescent religiosity. The final five classes solution is comprised of a 1) religiously disengaged class, 2) a religiously infrequent class, 3) a privately religious class, 4) a religious regulars class, and 5) a religiously devoted class. 1) The religiously disengaged class is characterized by an overall low level of engagement in religious activities and a low evaluation of the importance and influence of religious beliefs and friendships. 2) The religiously infrequent class is also characterized by an overall low level of engagement in religious activities but is more moderate in the assessment of the importance of religious beliefs. 3) The privately religious class is characterized by a low level of public religious engagement coupled with a very high evaluation of the importance and influence of religious beliefs and friendships. 4) The religious regulars class is characterized by moderate levels of public religious participation and a moderate to high evaluation of the importance and influence of religious beliefs and friendships. 5) The religiously devoted class is characterized by high levels of public religious participation and a very high evaluation of the importance and influence of religious beliefs and friendships. Being part of the religiously devoted class is protective against cocaine/crack use, with RR = 0.05, CI 95% 0.01–0.43, *p* < 0.05, and also being part of the religious regular class was protective (RR = 0.45, CI 95% 0.35–0.58, *p* < 0.05)	age, ethnicity, gender, family income, lifetime anxiety or depression, delinquency and violence	The protective influence of religiosity against cocaine use was not observed among privately religious youth who were strongly religious in terms of the valorization of religious beliefs and the influence of beliefs on decision-making yet religiously uninvolved in terms of participation in religious services and faith-based activities. These findings seem to suggest that, while private religiosity alone does not function as a protective factor, the combination of private religiosity with moderate to high public religious participation serves to effectively buffer young people from involvement in use of illicit substances
Wray-Lake et al., 2012 J Adolesc Health	Logistic regression models examined lifetime use of cocaine (including crack). After accounting for controls, religiosity was associated with lower odds of lifetime use of cocaine or crack (ORs = 0.57, *p* < 0.001)	gender, ethnicity, survey year, high school grades, parent’s education, social trust	Adolescents who reported greater social trust, social responsibility, and religiosity engaged in less use of cigarettes, alcohol, marijuana, and other illicit drugs, included cocaine/crack
Wallace & Bachman, 1991 Social Problems	Standardized regression coefficient for annual cocaine use and religiosity was—0.068 for women and −0.060 for men, *p* < 0.001	gender, ethnicity, parent’s education, urbanicity, college plans, truancy, employment, political views	Although truancy is overwhelmingly the most powerful correlate of cocaine use, evenings out, religious commitment, and living in the Northeast or West (relative to North Central) are also important correlates
Fothergill et al., 2009 J Health Soc Behav	Cocaine use and early adult church attendance coefficient in multivariate analyses was −0,107, *p* < 0,05	Gender, education, behavior, school achievement, economic resources, social bonds	For cocaine use, in multivariate analyses five variables had total effects equal to or above .10: early adult church attendance (− .107), early adult income (− .219), adolescent substance use (.214), first-grade aggression (.108), and first-grade shy behavior (− .100)

5 and

**Table 6:** Overview of studies measuring both religiosity and spirituality reviewed. Results

Author Year Journal	Results	Confounder	Conclusion
Allen & Lo, 2010 Journal of Drug Issues	At Stage 1 of the logistic regression, the crack cocaine use was regressed on the religiosity and control variables, and OR for religiosity and crack cocaine use was 0.958 (*b* = −0.043, but *p* > 0.05, so not statistically significant). At Stage 2 of the logistic regression, the spirituality variables were added, and OR for religiosity and crack cocaine use was 0.856 (*b* = −0.156, with a p < 0.05); OR for spirituality and crack cocaine use was 1.054 (*b* = 0.052, with *p* < 0.05). At Stage 3, with social bonding variables added, OR for religiosity and crack cocaine use was 0.863 (*b* = −0.148, with *p* < 0.05) and OR for spirituality and crack cocaine use was 1.053 (*b* = 0.051, *p* < 0.05)	age, gender, ethnicity, marital status, academic degree	While in the Stage 1 model religiosity was not found to be significant, at Stage 2 and Stage 3 religiosity exerted significant negative effects on crack cocaine use. In the Stage 2 model, both religiosity and spirituality demonstrated a significant relationship to lifetime crack cocaine use, religiosity as protective factor and spirituality as risk factor; the religiosity and spirituality variables continued to be significant in the Stage 3 model, maintaining the same directions, respectively. The negative relationship observed between religiosity and crack cocaine use was slightly reduced when social bonding variables were included in the Stage 3 model. This indicates that social bonding mediated religiosity’s impact on lifetime crack use
Watkins et al., 2016 J Relig Health	Religiosity was associated with cocaine use (*r* = 0.0978, *p* = 0.0010), and crack use (*r* = 0.1128, *p* = 0.0001). The spirituality index was negatively associated with cocaine use (*r* = −0.0719, *p* = 0.0153) and crack use (r = −0.1164, *p* = \0.0001)	ethnicity, age, HIV status, unprotected sexual intercourse	More religious Black MSM tended to report cocaine and crack use and were more likely to be HIV negative. Religiosity among Black MSM who use crack and cocaine was associated with higher levels of risky behavior as found in other studies and may increase risk of HIV and STD infection. Spirituality was negatively associated with unprotected anal sex, alcohol use, cocaine use, and crack use

6
